# Ocular Immersion Hydrotherapy Effectively Alleviate Symptoms and Signs of Severe Dry Eye Secondary to Ocular Graft‐Versus‐Host Disease in a Short Term

**DOI:** 10.1155/carm/8444085

**Published:** 2026-05-09

**Authors:** Haoran Cui, Shuang Zhang, Tao Zhang, Yong Tao

**Affiliations:** ^1^ Department of Ophthalmology, Beijing Chaoyang Hospital, Capital Medical University, Fengtai, Beijing, China, ccmu.edu.cn

**Keywords:** corneal epithelial cell damage, dry eye syndromes, graft vs host disease, hydrotherapy, ocular surface inflammation

## Abstract

**Purpose:**

To report a novel ocular immersion hydrotherapy (OIH) to manage patients with severe dry eye secondary to ocular graft‐versus‐host disease (oGVHD) and clinical outcomes after treatment.

**Methods:**

This case series included five consecutive patients with severe oGVHD‐related dry eye who had previously undergone unsuccessful long‐term conventional treatments. All five patients received an OIH treatment for a week. Visual acuity, Schirmer’s test (ST) without anesthesia, slit‐lamp examination, fluorescein corneal staining, and in vivo confocal microscopy were recorded before and after treatment. The corneal fluorescein staining was evaluated using the National Eye Institute (NEI) scale. The Ocular Surface Disease Index (OSDI) score was utilized to assess the severity of symptoms. Concentrations of IL‐2 and IL‐6 in tear fluid were quantified in another group of patients with oGVHD and dry eye disease (DED) at baseline, immediately following artificial tear instillation, and at 30 and 60 min postapplication.

**Findings:**

Following the OIH treatment in a short term, all patients exhibited significant improvement in both symptoms and signs, as evidenced by a substantial reduction in OSDI scores compared to pretreatment levels and a significant decrease in corneal fluorescein staining scores. In vivo confocal microscopy also indicated a reduction in inflammation. The concentrations of IL‐2 and IL‐6 in the tears of oGVHD patients are significantly higher than those in DED patients. Although artificial tear application transiently reduces the levels of both cytokines, they rebound to near‐baseline values within approximately 1 hour.

**Implications:**

Conventional treatments are often inadequate for managing severe and refractory dry eye secondary to oGVHD, leaving patients to suffer from severe symptoms. The novel OIH treatment approach presented in this study significantly promotes corneal epithelial healing, alleviates symptoms, and improves clinical signs, demonstrating good tolerability in a short term. Further long‐term studies with more patients are demanded.

## 1. Introduction

Graft‐versus‐host disease (GVHD) is a major complication of allogeneic hematopoietic stem cell transplantation (allo‐HSCT), a well‐established and potentially curative therapeutic approach for hematological disorders over the past few decades [1]. Chronic GVHD resembling autoimmune disorders occurs in 30%–70% of patients, leading to the involvement of multiple organ systems [2]. For instance, ocular GVHD (oGVHD) affects 40%–60% of allo‐HSCT patients and is more prevalent in those with chronic GVHD involving any organ system (60%–90%) [3]. oGVHD affects the ocular surface, lacrimal glands, and meibomian glands, resulting in instability of the tear film. The consequential visual impairment, pain, irritation, and other associated symptoms make oGVHD as a primary determinant of reduced quality of life [4].

Systemic immunosuppression for oGVHD is generally not recommended due to limited effectiveness and potential negative effects on graft‐versus‐tumor response and overall survival rates [5]. Topical management encompasses a range of strategies aimed at alleviating symptoms or maintaining the integrity of the ocular surface. These strategies include lubrication, tear preservation, prevention of tear evaporation, reduction of inflammation, support for the epithelium, implementation of supportive care measures, and surgical intervention [6]. However, the current therapeutic strategies for oGVHD still lack sufficient efficacy [4]. As the current management of oGVHD primarily relies on palliative measures based on tear substitutes, it is imperative to explore novel therapeutic interventions in order to enhance the prognosis of this debilitating condition.

In a previous study, we reported a case wherein the ocular immersion hydrotherapy (OIH) was utilized to successfully manage a refractory and severe dry eye condition secondary to oGVHD, which suggested that OIH could be a promising optional management for similar patients [7]. However, given that it was the first report of such a treatment protocol, its efficacy and safety require further validation through additional cases. This study aims to preliminarily assess the effectiveness and tolerability of OIH treatment approach for these patients with severe dry eye disease (DED) secondary to oGVHD.

## 2. Methods

To assess clinical efficacy and safety of OIH more comprehensively, we have implemented OIH therapy in subsequent consecutive 5 patients with severe DED secondary to oGVHD using swim goggles. During every single treatment session, patients wore swimming goggles disinfected with 75% alcohol. The goggle lenses were filled with a commercial electrolyte solution (Shike, Shenyang Xingqi Pharmaceutical Co., Ltd., Shenyang, China) to ensure that both eyes were fully immersed (Figure 1(a)). As per the instructions of the electrolyte solution, the primary constituents consist of sodium chloride, potassium chloride, magnesium sulfate, sodium bicarbonate, glucose, and calcium chloride (Figure 1(b)). After securing the head strap, patients wore the swim goggles continuously for 6 h. Schirmer’s test (ST) without anesthesia, slit‐lamp examination, corneal fluorescein staining (CFS), and in vivo confocal microscopy (IVCM) were employed to evaluate the patients’ ocular condition before and after treatment. The CFS was assessed using the National Eye Institute (NEI) scale, which assigns a score ranging from 0 (no staining) to 3 (heavy staining) for each of the five corneal regions (inferior, superior, central, nasal, and temporal) [8]. The total score of CFS, ranging from 0 to 15, represents the cumulative sum of all five areas. Scores of CFS before and after OIH treatment were analyzed by the Wilcoxon matched‐pairs signed rank test. The Ocular Surface Disease Index (OSDI) score was used to assess the severity of the patients’ subjective symptoms. It has been reported that interleukin‐2 (IL‐2) and interleukin‐6 (IL‐6) in tear cytokines are biomarkers for diagnosing oGVHD [9]. To provide a preliminary comparison of the benefits offered by OIH over frequent artificial tear administration, IL‐2 and IL‐6 concentrations in tear fluid were measured as reported using microsphere‐based immunoassay analysis (Solarbio, China) at baseline, immediately following artificial tear instillation, and at 30‐ and 60‐ minute intervals postapplication in a separate cohort comprising three oGVHD patients (6 eyes) and three DED patients (6 eyes). Statistical analyses were performed using Prism 9.5.0 (GraphPad, USA). *p* values < 0.05 were considered significant. This case series adheres to the guidelines for reporting uncontrolled case series [10].

FIGURE 1(a) Photograph of patient undergoing OIH treatment. (b) Schematic diagram of the OIH scheme. Main components of the electrolyte solutions contain sodium chloride, potassium chloride, magnesium sulfate, sodium bicarbonate, glucose, and calcium chloride.

**Figure:**
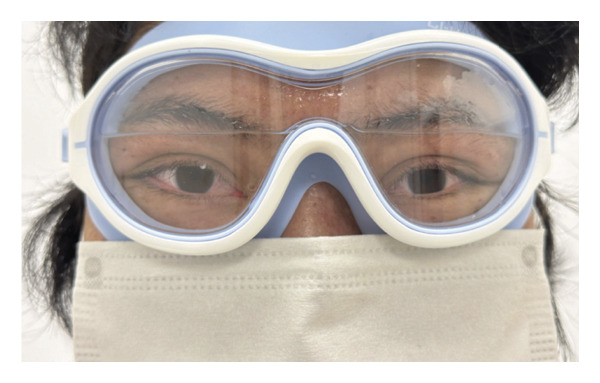
(a)

**Figure:**
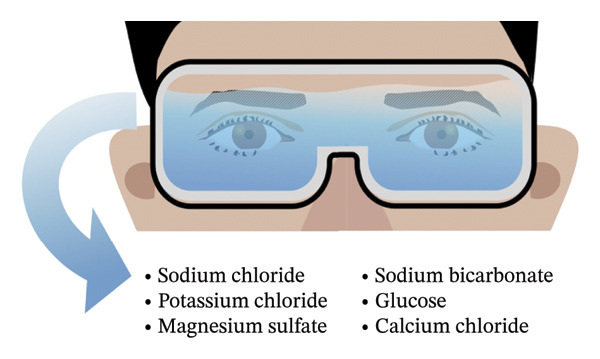
(b)

## 3. Case Presentation

**FIGURE 2:**
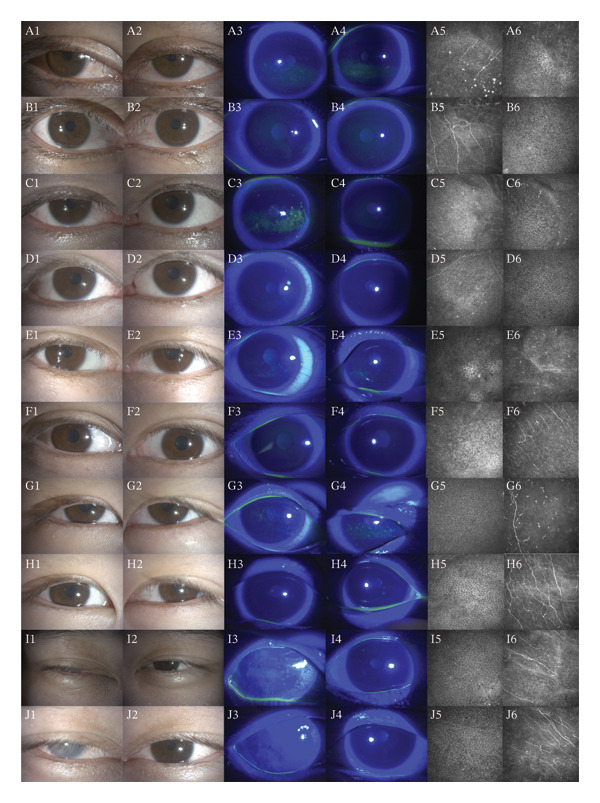
The data in each pair of consecutive rows in the figure represent pre‐ and post‐treatment information for a patient. The graph A‐B, C‐D, E‐F, G‐H, and I‐J correspond to Case 1‐5, respectively. The first, third, and fifth columns pertain to the patient’s right eye, while the second, fourth, and sixth columns correspond to the patient’s left eye. Figures 1‐2 depict slit‐lamp photographs of the patient, figures 3‐4 display corneal fluorescein staining results, and figures 5‐6 showcase in vivo confocal microscopy images.

**FIGURE 3:**
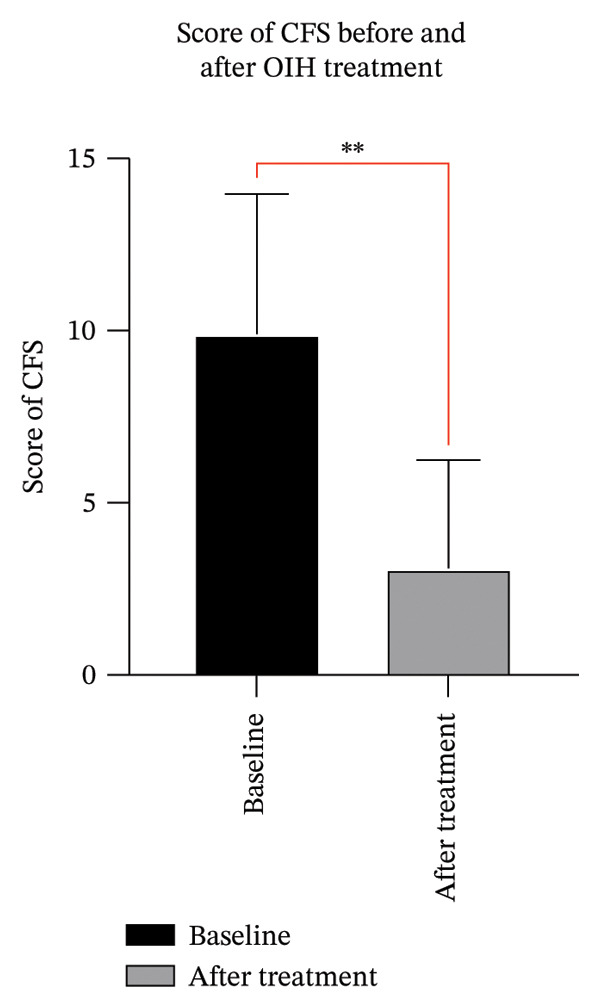
The pre‐ and post‐treatment CFS scores of 5 patients, revealing a notable reduction in CFS scores following treatment as compared to baseline, demonstrating statistical significance.

The slit lamp photographs, CFS, and IVCM images of five patients before and after treatment were integrated into a single composite image (Figure 2). The CFS scores of 10 eyes in 5 patients decreased significantly after treatment compared to the baseline level, with statistical significance (Figure 3). Certain patients demonstrated an enhancement in visual acuity following treatment (Figure 4(a)), while all patients displayed marked improvement in symptoms post‐treatment, as indicated by a decrease in OSDI scores (Figure 4(b)). In statistical analysis, the vision of HM/BE of Case 5 is recorded as 0.005 in decimal form [11]. The detailed content of the OSDI questionnaire of 5 patients is recorded in Table 1. The five cases are detailed in the following.

FIGURE 4(a) Mean BCVA of these 5 patients during their OIH treatment. Two of them got an improvement in vision acuity. Data are presented as decimal BCVA. (b) OSDI scores of these 5 cases before and after OIH treatment. OSDI scores decreased in all 5 cases after OIH treatment.

**Figure:**
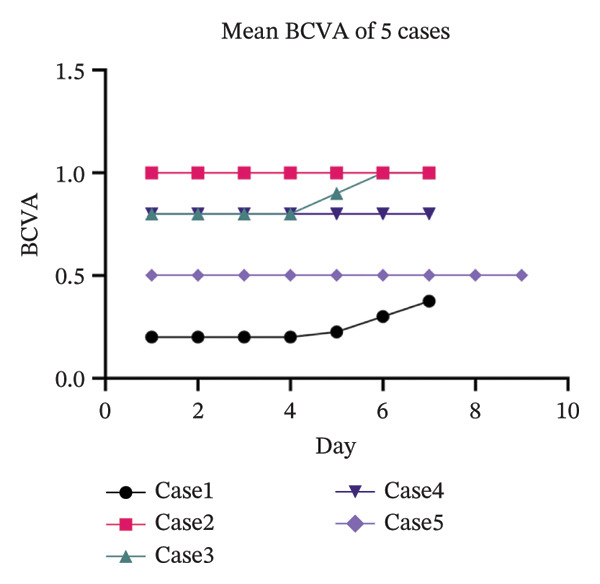
(a)

**Figure:**
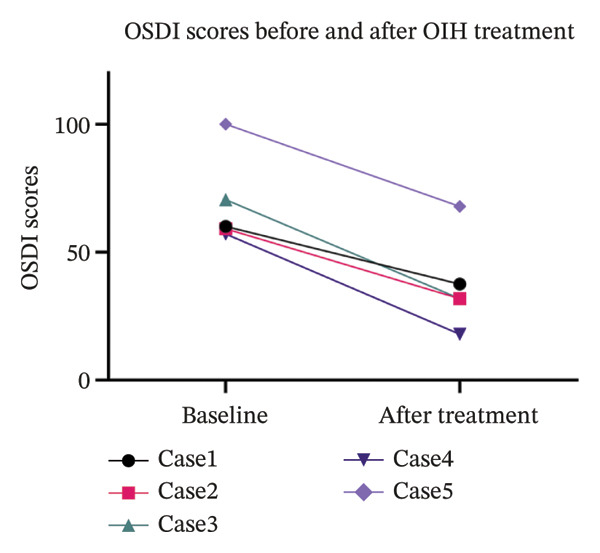
(b)

**TABLE 1:** OSDI scores of 5 cases with oGVHD.

	**Case 1**		**Case 2**		**Case 3**		**Case 4**		**Case 5**	
**Items**	**Baseline**	**AT**	**Baseline**	**AT**	**Baseline**	**AT**	**Baseline**	**AT**	**Baseline**	**AT**

1. Eyes that are sensitive to light	4	1	4	1	0	0	0	0	4	3
2. Eyes that feel gritty	1	1	4	2	4	2	4	1	4	2
3. Painful or sore eyes	1	0	3	1	3	1	4	1	4	2
4. Blurred vision	3	1	1	0	3	1	2	1	4	4
5. Poor vision	4	2	0	0	4	1	2	0	4	4
6. Reading	3	2	3	2	4	2	2	0	N/A	N/A
7. Driving at night	N/A	N/A	N/A	N/A	N/A	N/A	N/A	N/A	N/A	N/A
8. Working with a computer or ATM	3	2	2	2	3	1	N/A	N/A	N/A	N/A
9. Watching TV	N/A	N/A	2	2	3	1	2	2	N/A	N/A
10. Windy conditions	2	2	3	2	3	2	N/A	N/A	N/A	N/A
11. Places or areas with low humidity (very dry)	2	2	3	1	2	2	N/A	N/A	4	2
12. Areas that are air conditioned	1	2	1	1	2	1	N/A	N/A	4	2
OSDI score	60.0	37.5	59.1	31.8	70.5	31.8	57.1	17.9	100.0	67.9

*Note:* Score: 0 = none, 1 = rarely, 2 = sometimes, 3 = often, 4 = all the time.

Abbreviations: AT, after treatment; N/A, not applicable.

### 3.1. Case 1

A 26‐year‐old female patient primarily presented with photophobia accompanied by decreased visual acuity. Fourteen months ago, she was diagnosed with chronic myeloid leukemia and underwent a haploidentical allo‐HSCT at another hospital. Ten weeks post‐transplantation, she developed GVHD affecting her skin, oral cavity, and eyes, although she had no ocular symptoms at that time. The local hospital prescribed oral tacrolimus 0.25 mg daily for 3 months, which improved her GVHD, and she has continued on this medication since. Two months ago, she developed photophobia and decreased visual acuity in both eyes. The local hospital diagnosed her with ocular GVHD and initiated treatment with topical cyclosporine eye drops, fluorometholone eye drops, and sodium hyaluronate eye drops, each administered four times daily, with no significant improvement. One month ago, they added deproteinized calf blood extract ophthalmic gel, but the patient reported minimal symptomatic relief and subsequently sought treatment at our hospital. The patient had refractive errors of −2.0 DS in the right eye and −1.75 DS in the left eye.

Upon admission, her BCVA was 20/100 in both eyes, with intraocular pressures of 16 mmHg in the right eye and 14 mmHg in the left eye. A slit‐lamp examination revealed eyelid edema, mild conjunctival hyperemia, and corneal epithelial defects in both eyes (Figure 2 A1‐4). IVCM indicated an increased presence of inflammatory cells in the corneas of both her eyes and a reduction of nerve fiber (Figure 2 A5‐6). ST without anesthesia measured less than 5 mm in both eyes. Her OSDI score was 60 when she was admitted to hospital.

Following admission, she received 6 hours of daily OIH treatment during the daytime. She was instructed to use ofloxacin eye ointment once daily after completing her daily OIH treatment sessions. No additional topical treatments were administered.

After 1 week of OIH treatment, her BCVA improved to 20/20 in both eyes. Intraocular pressure was 14 mmHg in the right eye and 15 mmHg in the left eye. There was a marked reduction in upper eyelid edema and conjunctival hyperemia, with near‐complete resolution of corneal epithelial defects in both eyes (Figure 2 B1‐4). IVCM indicated an increase in epithelial cell density, improved epithelial integrity, reduced inflammatory infiltration, and increased nerve plexus density (Figure 2 B5‐6). ST without anesthesia measured 6 mm in both eyes. The patient reported a near‐complete resolution of photophobia and significant improvement in visual acuity, with an OSDI score of 37.5 at discharge.

### 3.2. Case 2

A 21‐year‐old male patient primarily presented with a 1‐year history of dry eyes and photophobia, with more severe symptoms in the right eye, including a foreign body sensation. The patient underwent Trans‐PRK refractive surgery for myopia correction 4 years ago. One and a half years ago, he was diagnosed with acute B‐cell lymphoblastic leukemia and underwent an allogeneic fully matched hematopoietic stem cell transplantation at another hospital. Post‐transplantation, he has been on olverembatinib 40 mg every other day. One year ago, he developed GVHD affecting the skin, oral cavity, and eyes. Although his condition improved following anti‐rejection treatment, he continued to experience persistent dry eyes and photophobia, with more severe symptoms and a foreign body sensation in the right eye. The local hospital’s treatment regimen included sodium hyaluronate eye drops three times daily and ofloxacin eye ointment at bedtime, but his symptoms did not improve, prompting him to seek treatment at our hospital.

His BCVA upon admission was 20/20, with intraocular pressure of 15 mmHg in both eyes. Slit‐lamp examination revealed eyelid edema and conjunctival hyperemia in both eyes, with more severe findings in the right eye. CFS showed significant epithelial damage in the right eye and slight corneal epithelial defects in the left eye (Figure 2 C1‐4). IVCM revealed inflammatory cells characterized by numerous highly reflective dots. Additionally, there was a concurrent decrease in epithelial cell density and disruption of epithelial integrity (Figure 2 C5‐6). ST without anesthesia measured less than 5 mm in the right eye and 5 mm in the left eye. His OSDI score was 59, primarily due to severe grittiness and photophobia.

He underwent 6‐h‐per‐day treatment of OIH for both eyes in the day time. Artificial tears could be used once before and after OIH treatment in the day time. He continued to use ofloxacin eye ointment at bedtime just as he did before being admitted for treatment. No additional topical medications were administered.

After 1 week of treatment, his BCVA remained unchanged at 20/20 in both eyes, with intraocular pressure remaining at 15 mmHg. Both eyes exhibited improvements in eyelid edema and conjunctival hyperemia, with more pronounced effects in the right eye. Although the corneal epithelial damage in the right eye did not completely resolve, it showed significant improvement (Figure 2 D1‐4). IVCM indicated a marked improvement in epithelial integrity and clear visualization of subbasal nerves in the right eye (Figure 2 D5‐6). ST without anesthesia measured 5 mm in both eyes. The patient reported almost complete resolution of the foreign body sensation in the right eye and significant relief from dry eye and photophobia symptoms in both eyes. His OSDI score was reduced to 31.8.

### 3.3. Case 3

A 29‐year‐old female patient primarily presented with newly onset dry eyes, foreign body sensation, and decreased visual acuity. She has a 15‐year history of hepatitis B, for which she has been taking entecavir and adefovir dipivoxil. The patient has no other systemic diseases. Nine months ago, she was diagnosed with acute myeloid leukemia (M2) and underwent a haploidentical allo‐HSCT at another hospital. She is currently on maintenance therapy with gilteritinib. Four months ago, she developed GVHD affecting her skin and eyes. Following anti‐rejection treatment, her condition improved, but she continued to experience persistent symptoms of dry eyes, foreign body sensation, and decreased visual acuity. The local hospital’s treatment regimen included sodium hyaluronate eye drops administered 6–8 times daily, tobramycin‐dexamethasone eye drops 3 times daily, and tobramycin‐dexamethasone eye ointment at bedtime, along with cold compresses. However, her symptoms did not improve. Subsequently, the local hospital added topical tacrolimus eye drops, but she discontinued it due to a burning sensation and sought treatment at our hospital.

Upon admission, ophthalmic examination revealed BCVA of 20/20 in the right eye and 20/33 in the left eye. Intraocular pressure was 13 mmHg in the right eye and 15 mmHg in the left eye. Compared to other patients, she exhibited less pronounced eyelid edema and conjunctival hyperemia, but both eyes had extensive corneal epithelial damage (Figure 2 E1‐4). IVCM indicated disrupted epithelial integrity and significant inflammatory infiltration (Figure 2 E5‐6). ST without anesthesia was 3 mm in both eyes. The OSDI score was 70 before OIH treatment.

The patient underwent 6‐h‐per‐day treatment of OIH for both eyes in the day time. Artificial tears could be used as needed before initiating treatment in the morning. Tobramycin‐dexamethasone eye ointment was still used at bedtime as before. No additional topical medications were administered.

After 7 days of treatment, the BCVA in her right eye remained stable at 20/20, while the BCVA in her left eye improved to 20/20. Intraocular pressure was at 13 mmHg in both eyes. There was a reduction in upper eyelid edema, and the palpebral fissure width increased (Figure 2 F1‐2). The corneal epithelium in both eyes nearly completely healed (Figure 2 F3‐4). IVCM demonstrated reduced inflammatory cell infiltration and increased nerve plexus density (Figure 2 F5‐6). Despite the ST without anesthesia remaining at 3 mm in both eyes, the patient reported satisfaction with the improvement in her visual acuity. Additionally, her OSDI score decreased to 31.8.

### 3.4. Case 4

A 7‐year‐old female patient primarily presented with dry eyes and difficulty in opening her eyes for three and a half years. Four years ago, she was diagnosed with acute megakaryoblastic leukemia (M7) and underwent a haploidentical allo‐HSCT at another hospital. Postoperatively, she developed GVHD affecting the skin, gastrointestinal tract, and eyes. Following treatment for GVHD, her condition improved, but symptoms of dry eyes and difficulty opening her eyes persisted. The treatment regimen at the local hospital included topical application of artificial tears, fluorometholone, and deproteinized calf blood extract eye gel, each administered four times daily. During the treatment period, her symptoms of dry eyes intermittently improved, but she continued to experience persistent difficulty in opening her eyes.

Upon admission, the patient’s visual acuity was 20/25 in both eyes. Similar to the previous adult patient, this patient also presented with eyelid edema, mild conjunctival hyperemia, and patchy corneal epithelial damage (Figure 2 G1‐4). The inflammatory cells presented with several high‐reflective dots or branches near the Bowman’s membrane by IVCM, with nerve plexus density significantly lower than normal (Figure 2 G5‐6). ST without anesthesia was 6 mm in both her eyes. And the OSDI score of this patient was 57.1.

After 1 week of treatment, her visual acuity remained 20/25, but her difficulty in opening her eyes markedly improved. The palpebral fissure width in the right eye increased, while there was no significant change in the left eye (Figure 2 H1‐2). Although she was unable to fully cooperate with the CFS, preventing complete visualization of the cornea, the visible areas showed nearly complete healing of the corneal epithelial damage that was present pretreatment (Figure 2 H3‐4). IVCM demonstrated a near absence of inflammatory cells and a significant improvement in nerve plexus density (Figure 2 H5‐6). The improvement of the subjective symptoms was also very obvious, and her OSDI score decreased to 17.8.

### 3.5. Case 5

The patient, a 19‐year‐old male, reported experiencing dry eyes, photophobia, and a grittiness in both eyes for two and a half years. Four years ago, he was diagnosed with acute lymphoblastic leukemia (B‐ALL) and underwent a haploidentical hematopoietic stem cell transplantation (father‐to‐son) at another hospital. Two and a half years ago, he began experiencing dry eyes and photophobia, with intermittent grittiness in both eyes. The local hospital diagnosed him with GVHD and administered topical treatments including artificial tears and fluorometholone eye drops, which were ineffective. Subsequently, the patient underwent amniotic membrane transplantation for corneal perforation and dissolution in the right eye, as well as two conjunctival flap covering surgeries and eyelid margin suturing. Two years ago, due to uncontrollable corneal perforation, he underwent lamellar keratoplasty in the right eye at a local hospital. Postsurgery, he was treated with artificial tears, fluorometholone eye drops, tacrolimus eye drops, and cyclosporine eye drops, but the symptoms of dry eyes and eye pain persisted. One month ago, he underwent conjunctival flap covering surgery for a corneal ulcer in the right eye again. His topical treatment regimen included artificial tears every half hour, fluorometholone eye drops, tacrolimus eye drops, and cyclosporine eye drops four times per day, yet he continued to experience dry eyes, eye pain, foreign body sensation, and photophobia.

Upon admission, his right eye vision acuity was HM/BE (hand movement before eye), with significant eyelid skin edema, almost preventing voluntary eye opening, with mixed conjunctival hyperemia in the right eye and extensive patchy fluorescein staining on the graft surface (Figures 2, 11, I3). His left eye vision acuity was 20/20, with eyelid skin edema, slight conjunctival hyperemia, and slight dot‐like corneal erosion (Figure 2 I2, I4). IVCM could not capture images of the right eye, but the left eye showed minimal inflammatory cell infiltration (Figurse 2 I5‐6). His OSDI score at admission was 100, and he refused to undergo ST.

He conducted 9 consecutive days of OIH treatment in the day, with each session lasting 6 h. Given his postkeratoplasty status and corneal ulcers, levofloxacin eye drops were added to the electrolyte solution during each OIH session to prevent potential infection risks.

After 9 days of treatment, his vision remained unchanged in both eyes, but his eyelid edema in both eyes significantly reduced, allowing voluntary eye opening. The degree of hyperemia in the right eye decreased to a level similar to that of the left eye, and the fluorescein staining on the graft surface nearly completely resolved (Figure 2 J1‐4). More subbasal nerves could be clearly detected in IVCM, and the density of the nerve plexus was also improved (Figure 2 J5‐6). Besides, his subjective symptoms improved markedly, with his OSDI score reducing to 67.8.

### 3.6. Concentrations of Cytokines in Tear Fluid

Following screening, both the oGVHD and DED groups included 3 participants (6 eyes each). There were no statistically significant differences in age or gender between the two groups; however, significant differences were observed in OSDI scores, TBUT, and CFS, as shown in Table 2.

**TABLE 2:** Age and ocular surface parameters of oGVHD and DED patients.

	**oGVHD**	**DED**	**p**

Age	37.67 ± 16.80	29.33 ± 9.29	0.49
OSDI score	79.38 ± 16.11	28.35 ± 11.60	0.01
Fluorescein tear film break‐up time (sec)	0.50 ± 0.55	3.67 ± 0.82	< 0.0001
Schirmer’s tear secretion score (mm)	1.17 ± 0.98	9.33 ± 1.63	< 0.0001
Corneal fluorescein staining score	11.83 ± 2.04	0.50 ± 0.55	< 0.0001

Analysis of tear IL‐2 and IL‐6 concentrations revealed significantly higher levels in oGVHD patients compared to DED patients. This aligns with previous literature, validating the reliability of the measurements. Immediately after the administration of artificial tears, cytokine concentrations in the conjunctival sac of oGVHD patients decreased but gradually rebounded over time, approaching baseline levels at 60 min, as illustrated in Figure 5.

FIGURE 5IL‐2 (a) and IL‐6 (b) levels in oGVHD patients were higher than in DED patients. Postinstillation of artificial tears, concentrations dropped below baseline but gradually recovered to near‐baseline levels.

**Figure:**
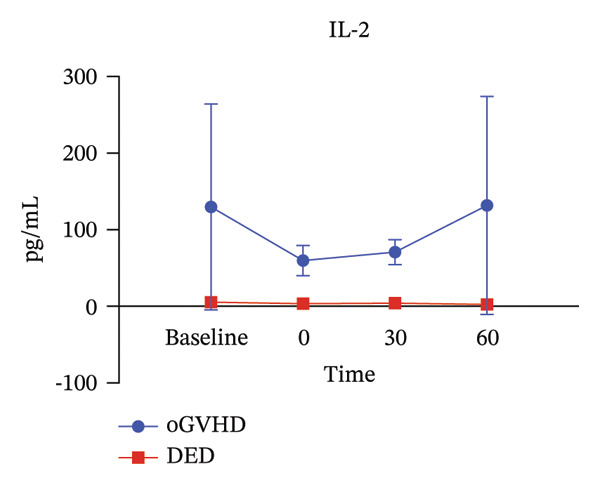
(a)

**Figure:**
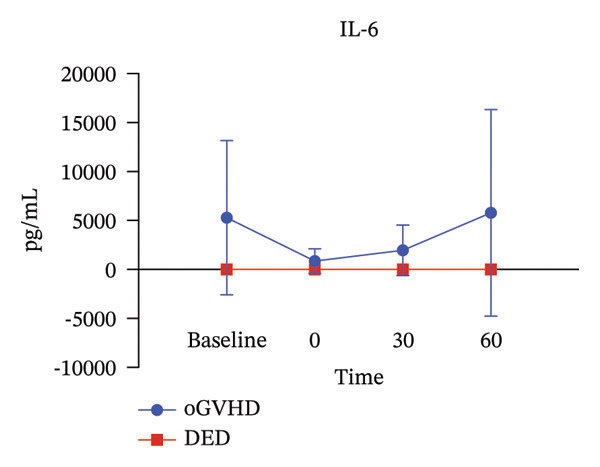
(b)

## 4. Discussion

Corneal damage is a prominent pathological mechanism in oGVHD, which causes pain, photophobia, vision impairment, and high‐frequency application of tear substitutes, significantly affecting the patient’s activities of daily living [4]. Currently, the primary management objectives for DED or oGVHD are to control symptoms and signs by enhancing tear function and neural health [4, 12]. A significant portion of topical treatments targets the health of the tear film and epithelial health, aiming to alleviate patient symptoms and reduce discomfort [13]. Consequently, promoting corneal epithelial healing may be a crucial aspect of oGVHD treatment.

The concept of moist healing, which was first introduced by George Winter in 1962 [14], has gained wide acceptance. Extensive research has provided ample evidence that a moist environment significantly accelerates the migration rate of epidermal cells, accelerating the epithelialization process [15–18]. This concept inspired us to explore the use of OIH to promote corneal epithelial healing as a treatment for oGVHD. During OIH treatment, the ocular surface is maintained in a moist environment by the electrolyte solution, contrasting sharply with the pathological state of tear deficiency in oGVHD or DED. In the initial case and the 5 patients reported herein, corneal epithelial defects showed significant improvement in the moist environment, as evidenced by post‐treatment CFS (Figure 3). The healing of the corneal epithelium not only greatly alleviated symptoms of dryness and foreign body sensation but also markedly reduced photophobia and vision deterioration, as reflected in the decreased OSDI scores of the five patients post‐treatment (Figure 4).

Furthermore, oGVHD patients widely use artificial tears, which may act by diluting inflammatory factors on the ocular surface, thereby protecting corneal epithelial health [13]. Theoretically, frequent application of artificial tears could dilute inflammatory cytokines on the ocular surface; however, this feasibility appears to be limited to patients with conventional dry eye. As shown in Figure 5, likely due to the higher baseline levels of ocular surface inflammation in oGVHD patients, the dilution effect of artificial tears dissipates rapidly. To effectively dilute these cytokines, oGVHD patients would require a much higher frequency of instillation, which increases treatment burden, reduces compliance, and negatively impacts quality of life. In contrast, in OIH treatment, this diluting mechanism might be amplified due to the increased volume of the solution, reducing ocular surface inflammation. Post‐treatment photographs of the patients showed varying degrees of relief in conjunctival hyperemia and eyelid edema, which were further confirmed by IVCM.

The five patients in this report included those with corneal ulcers postkeratoplasty and a seven‐year‐old child. All patients reported that during their hospitalization for OIH treatment, dryness and gritty sensation almost completely disappeared while wearing swimming goggles, with no other discomforts except occasional skin pressure from prolonged use. No ocular infections occurred during or after OIH treatment. Therefore, the observed cases suggest that OIH therapy is safe and well‐tolerated.

To alleviate discomfort when not wearing swimming goggles, some patients used artificial tears or ointments, with the same compositions as before admission and in lower doses. No additional systemic or local steroids or immunosuppressants were added. Thus, the reduction in symptoms and improvement in signs can primarily be attributed to the effects of OIH therapy. Nevertheless, OIH treatment was performed for the 5 patients within only 1 week. Long‐term effects and complications of OIH remains unknown, and further studies are demanded.

## 5. Conclusions

OIH may serve as a short‐term therapeutic option for patients with severe and refractory dry eye secondary to oGVHD, potentially promoting corneal epithelial healing and alleviating symptoms.

## Author Contributions

Haoran Cui: data curation; investigation; methodology; visualization; and writing–original draft. Shuang Zhang: data curation and investigation. Tao Zhang: writing–review and editing. Yong Tao: conceptualization; methodology; and writing–review and editing.

## Funding

This study was supported by the Beijing Hospitals Authority’s Ascent Programme, Capital Health Development Scientific Research Project (Grant No. 2022‐2–2035), Beijing Nova Program (No. 20230484445), and Excellent Young Talent Innovation Project (CX23YQ03 and CX23YQA02).

## Ethics Statement

This study conformed to the provisions of the Declaration of Helsinki and was approved by the Institute Review Board (IRB) and Ethics Committee of Beijing Chaoyang Hospital, Capital Medical University (LGH‐2024–04–019). Written informed consent was obtained from the patient prior to the treatment of this case report that their information would be used in the case report.

## Conflicts of Interest

The authors declare no conflicts of interest.

## Data Availability

Data are available on request from the authors.
